# Dual-Hydrogen-Bonded Interfaces Unlock Record-High Efficiency and Stability in Eco-friendly Copper-Iodide Deep-Blue Light-Emitting Diodes

**DOI:** 10.34133/research.0935

**Published:** 2025-10-07

**Authors:** Ting Pan, Yu Shen, Tangzhe Chen, Rui Xu, Wen-Yong Lai

**Affiliations:** State Key Laboratory of Flexible Electronics (LoFE), Institute of Advanced Materials (IAM), School of Chemistry and Life Sciences, Nanjing University of Posts & Telecommunications, Nanjing 210023, China.

## Abstract

Eco-friendly deep-blue light-emitting diodes (LEDs) with excellent efficiency and stability represent a critical bottleneck for next-generation displays. Zhu et al., reporting in *Nature*, present a copper-iodide hybrid emitter achieving record performance via dual interfacial hydrogen-bond passivation. By sandwiching the copper-iodide emissive layer between a self-assembled monolayer and a poly(methyl methacrylate) layer, this approach effectively passivates interfacial defects, facilitates hole injection, and suppresses excess electron transport. The optimized LEDs demonstrate a 12.57% external quantum efficiency, a 3,970 cd m^−2^ luminance (CIE: 0.147, 0.091), and a 204-h half-lifetime at 100 cd m^−2^, representing a stability improvement exceeding 85-fold over those of perovskite counterparts. This work not only delivers unprecedented efficiency and an operational lifetime for metal halide deep-blue LEDs but also establishes a dual-interfacial-hydrogen-bond-passivation-based design principle for advancing sustainable displays.

Light-emitting diodes (LEDs) represent a class of highly efficient solid-state emitters that are fundamentally transforming the fields of illumination and full-color display technology. Their key technological applications encompass flexible screens, ultrafast data transmission, and high-capacity information storage [1]. Substantial progress has been made over the last 10 years, with solution-processed luminescent materials designed for green-, red-, and near-infrared-emitting LEDs now demonstrating external quantum efficiencies (EQEs) surpassing 20% [2–5]. Achieving the Rec. 2020 color gamut standard necessitates deep-blue emitters requiring a Commission Internationale de l’Éclairage *y* coordinate (CIE*_y_*) ≤0.06 and an emission wavelength ≤465 nm. While organic semiconductors (platinum complexes, 464 nm, EQE 30.8%, *T*_50_ = 670 h at 1,000 cd cm^−2^) [6], quantum dots (ZnSeTeS/ZnSe/ZnS, 460 nm, EQE 24.7%, *T*_50_ = 30,000 h at 100 cd cm^−2^) [7], lead-based perovskites (459 nm, EQE 15.36%, *T*_50_ = 2.4 h at 20 cd cm^−2^) [8], and lead-free perovskites (CsEuBr_3_, 448 nm, EQE 6.5%, *T*_50_ = 0.8 h at 20 cd cm^−2^) [9] can be compositionally engineered to emit deep-blue light, their practical application is significantly limited by complex fabrication processes, high costs, toxic heavy metals, and poor operational stability. Thus, there is an urgent demand for eco-friendly alternatives that exhibit comparable efficiency, excellent color fidelity, and improved operational stability [10–12].

Recently, Zhu et al. [13] reported a breakthrough in solution-processable copper-iodide hybrids using Cu_4_I_8_(Hdabco)_4_ (Hdabco = 1,4-diazabicyclo-[2.2.2]octane-1-ium), achieving an EQE of 12.57% and a *T*_50_ of 204 h (at 100 cd m^−2^). The crystal structure comprises zigzag one-dimensional (Cu_4_I_8_)^4−^ inorganic chains linked to (Hdabco)_2_^2+^ dimers via coordinate and ionic bonds (Fig. 1A). This one-dimensional architecture confers enhanced structural rigidity, long Cu–Cu distances (>3.3 Å), a direct bandgap of 3.8 eV, and enhanced exciton confinement due to high-lying ligand energy levels, distinguishing it from zero-dimensional copper-halide clusters [14]. Spin-coated thin films exhibit pinhole-free morphology, low surface roughness (*R*_a_ = 0.177 nm), high crystallinity, and preferential (200) orientation. These films achieve a near-unity photoluminescence quantum yield of 94.7% and intense deep-blue emission at 449 nm (CIE: 0.147, 0.087). Strong spin–orbit coupling, a small singlet–triplet gap (Δ*E*_S1-T1_ = 50.8 meV), and delocalized electronic states near the conduction band minimum enable multichannel radiation: fluorescence, thermally activated delayed fluorescence, and dominant phosphorescence. Space-charge-limited-current measurements reveal balanced carrier mobilities (hole and electron mobilities of 5.9 × 10^−4^ and 8.8 × 10^−4^ cm^2^ V^−1^ s^−1^, respectively) and a low trap density (8.2 × 10^16^ cm^−3^), rivaling those of high-quality perovskites and surpassing those of conventional copper halides [15,16].

**Fig. 1.**
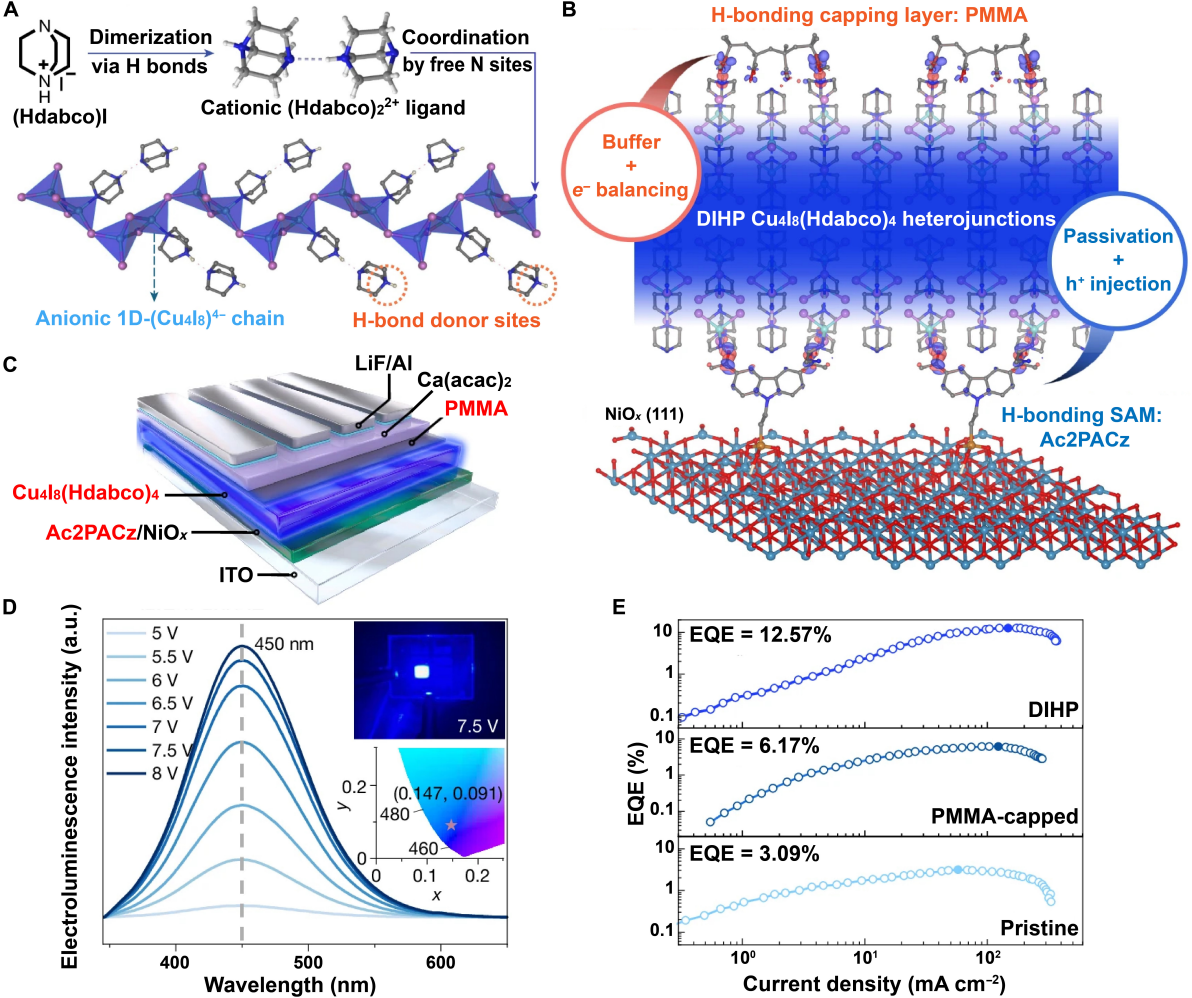
(A) Structural building blocks of Cu_4_I_8_(Hdabco)_4_ (Hdabco = 1,4-diazabicyclo-[2.2.2]octane-1-ium). (B) Schematic diagram of the dual interfacial hydrogen-bond passivation (DIHP) approach applied to Cu_4_I_8_(Hdabco)_4_. (C) Device schematic of the fabricated deep-blue light-emitting diode (LED). (D) Electroluminescence spectra of the LED device operated under 5 to 8 V. Inset: photograph of the DIHP LED under 7.5 V (top) and the coordinates of the luminescence in CIE 1931 color-space system (bottom). (E) Comparative plots of the external quantum efficiency (EQE)–current density curves of various LEDs. Reproduced with permission [13]. Copyright 2025, Springer Nature. 1D, one-dimensional; PMMA, poly(methyl methacrylate); SAM, self-assembled monolayer; Ac2PACz, 2-(3,6-diacetyl-9*H*-carbazol-9-yl)ethyl phosphonic acid; ITO, indium tin oxide.

To mitigate interfacial losses arising from trap-assisted recombination and charge imbalance, Zhu et al. introduced a novel dual interfacial hydrogen-bond passivation (DIHP) approach (Fig. 1B). Specifically, a self-assembled monolayer (SAM) composed of Ac2PACz (2-(3,6-diacetyl-9*H*-carbazol-9-yl)ethyl phosphonic acid) was deposited at the interface between the hole transport layer and the emissive layer. With this SAM, phosphonic acid anchoring groups effectively mitigate trap states (6.3 × 10^16^ cm^−3^, 3.6-fold lower than that in the untreated device), while the carbazole moieties function to reduce the hole mobility (9.0 × 10^−4^ cm^2^ V^−1^ s^−1^, 1.5-fold higher than that of the untreated device). Conversely, at the emissive layer/electron transport layer junction, an extremely thin poly(methyl methacrylate) (PMMA) layer was inserted. This polymer dielectric serves a dual purpose: modulating the electron injection flux and passivating interfacial defects through the formation of carbonyl–amine hydrogen bonds. First-principles calculations confirm strong hydrogen-bond energy at both interfaces (2.99 eV for Ac2PACz–Cu_4_I_8_(Hdabco)_4_; 1.37 eV for PMMA–Cu_4_I_8_(Hdabco)_4_), facilitating charge-density relocation and minimizing nonradiative losses. As detailed in Fig. 1C to E, the device architecture (indium tin oxide/Ac2PACz–NiO*_x_*/Cu_4_I_8_(Hdabco)_4_/PMMA/Ca(acac)_2_/LiF/Al) delivered outstanding performance metrics: a deep-blue emission at 450 nm (CIE: 0.147, 0.091), a maximum luminance reaching 3,970 cd m^−2^, an EQE of 12.57%, and a *T*_50_ of 204 h (unencapsulated, 100 cd m^−2^). The EQE of the DIHP-modified device was nearly 2-fold and 4-fold higher than those of the device with only PMMA capping and the untreated device, respectively. This result clearly confirms the synergistic advantages of DIHP—i.e., the combined effects of dual interfacial passivation and the electron-blocking role of the PMMA layer—over single-interface or nonpassivation schemes. Remarkably, this *T*_50_ value exceeds that of comparable perovskite-based LEDs by more than 85 times. Furthermore, scalability was demonstrated through a larger 4-cm^2^ device, which maintained a respectable EQE of 7.87%.

This study presents a nontoxic, bright emitter and an effective fabrication method for robust deep-blue LEDs. Critically, it highlights the advantages of a dual-interface synergistic modulation strategy over conventional single-interface passivation approaches, demonstrating superior optimization of dynamic charge injection and device performance. Despite these advances, the current device performance exhibits gaps relative to the Rec. 2020 color gamut standard, necessitating targeted improvements in 3 key areas: (a) Photometric parameter optimization: Narrow the emission bandwidth via ligand engineering—specifically, by introducing conjugated rigid moieties (e.g., benzene, carbazoles, and triazine rings) to enhance ligand steric hindrance, suppress lattice vibrations, and regulate exciton diffusion. Additionally, incorporating heavy atoms will strengthen spin–orbit coupling to boost phosphorescence efficiency. To further improve device metrics, precise control over the crystallinity and orientation of copper-iodide films, combined with electrode optimization, will enhance charge mobility and reduce operating voltage. (b) Interfacial engineering advancement: Facilitate efficient hole injection by modifying carbazoles with electron-donating/electron-withdrawing substituents to enhance intramolecular charge transfer. Expand the DIHP strategy by developing novel hydrogen-bonding materials, including fluorene-based molecules and carbonyl/carboxyl-functionalized polyimides. (c) Degradation mechanisms’ elucidation [11]: Employ in situ characterization techniques to clarify the correlation between copper-iodide structural degradation, interface deterioration, and efficiency roll-off. Key research priorities include investigating copper-iodide ion migration, hydrogen-bond dissociation in SAM and polymer layers, defect regeneration, energy level misalignment, and charge injection imbalance. Although copper-iodide-based LEDs are an emerging field, the approach presented here, which effectively passivates defects and optimizes charge injection in hybrid emitters, offers important potential for advancing deep-blue LED research. The molecular design concepts demonstrated in Zhu et al.’s work—specifically, the design of bifunctional cationic ligands and strong hydrogen-bonding passivation layers—hold broad applicability beyond copper-iodide LEDs. These principles can be extended to other optoelectronic devices, including perovskite LEDs, quantum dot LEDs, organic LEDs, and even photovoltaics. By adapting ligand functionality and hydrogen-bonding interactions to match the unique needs of each device system, researchers can potentially achieve marked improvements in both efficiency and operational lifetime across multiple optoelectronic fields.
